# Effects of cold air inhalation on body temperature, respiratory and cerebrovascular responses during exercise in the heat

**DOI:** 10.1186/2046-7648-4-S1-A128

**Published:** 2015-09-14

**Authors:** Bun Tsuji, Yorinobu Chinda, Yasushi Honda, Naoto Fujii, Narihiko Kondo, Takeshi Nishiyasu

**Affiliations:** 1 Research Fellow of Japan Society for the Promotion of Science, University of Tsukuba, Japan; 2 Institute of Health and Sport Sciences, University of Tsukuba, Japan; 3 Faculty of Human Development, Kobe University, Japan

## Introduction

Hyperthermia during exercise leads to increases in ventilation independently of metabolic factors, resulting in hypocapnia and cerebral hypoperfusion [1], which is one of the mechanisms behind impaired exercise performance in the heat. To suppress hyperthermia, cold water immersion and ingestion of cold drinks are commonly used, but the effect of cold air inhalation on physiological responses during hyperthermia is not well understood. This study examined the effects of cold air inhalation on body temperature, respiratory and cerebrovascular responses during exercise in the heat.

## Method

Twelve male subjects [age 24 ± 4 years, height 174 ± 4 cm, weight 70 ± 4 kg, peak oxygen uptake (VO_2peak_) 48.5 ± 6.5 mL.kg^-1^.min^-1^] performed a cycle exercise at 50% of VO_2peak _in the heat (38 °C ambient temperature and 50% relative humidity) until their esophageal temperature (T_es_) reached 39 °C or they could no longer continue the exercise. Throughout the exercise on two separate occasions, subjects inhaled room air (i.e., 38 °C; Hot-air trial) or cold air (10 °C; Cold-air trial). T_es_, minute ventilation, respiratory gases, sweat rate (ventilated capsule method) and skin blood flow (laser-Doppler) on the chest, middle cerebral artery blood velocity (transcranial Doppler ultrasound) and arterial blood pressure were measured continuously.

## Results

Exercise duration was higher in the Cold- than Hot-air trial (57.1 ± 13.7 vs. 45.8 ± 6.7 min, *P *< 0.01). T_es _was lower in the Cold- than Hot-air after 35 min of exercise (*P *< 0.01). Cutaneous vascular conductance (skin blood flow/mean arterial pressure) and VO_2 _did not differ between trials (*P *= 0.57 and 0.22, respectively), but sweat rate was lower in the Cold-air trial (*P *= 0.032). Minute ventilation was lower (*P *= 0.011) and estimated PaCO_2 _was higher (*P *= 0.015) in the Cold- than Hot-air trial. Ventilatory sensitivity to rising T_es _(slope of the T_es_-ventilation relation) was similar between Hot- and Cold-air trials (10.3 ± 7.7 vs. 10.7 ± 9.2 L.min^-1^.°C^-1^, *P *= 0.71). Cerebral vascular conductance (middle cerebral artery blood velocity/mean arterial pressure) was higher in the Cold-air trial (*P *= 0.049).

## Discussion

Consistent with a previous study in which cold air inhalation during hyperthermic exercise decreased core temperature mainly due to increases in respiratory heat exchange (2), we found lower T_es _in the Cold-air trial. We also found that cold air inhalation induced the lower ventilation but similar ventilatory sensitivity to rising T_es _compared to Hot-air. These suggest that the lower ventilation during cold air inhalation was solely due to decreases in T_es_. In addition, it was reported that reduction in cerebral blood flow velocity during exercise in the heat is largely accounted for by the hyperventilation-induced decrease in PaCO_2 _(3). Thus, the increases in cerebral vascular conductance in the Cold-air trial was likely attributable to cold air inhalation-induced suppressions of hyperventilation and hypocapnia.

## Conclusion

Present results indicate that during prolonged exercise in the heat, cold air inhalation mitigates changes in core temperature, ventilation and cerebral blood flow.
